# Supplementary material for: The Effect of Physiotherapy Group Intervention in Reducing Pain Disorders and Mental Health Symptoms among Syrian Refugees: A Randomized Controlled Trial
Source: Int J Environ Res Public Health. 2020 Dec 17;17(24):9468. doi: 10.3390/ijerph17249468 (PMC7767069; doi:10.3390/ijerph17249468)
Supplement: Supplementary file 1 [file ijerph-17-09468-s001.pdf]

Supplementary Table S1. Number of participants who attended physiotherapy activity and awareness intervention (PAAI) sessions in each intervention and control group, by gender.

Type of intervention	TOTAL *n*	S1(%) (Q1a)	S2 (%)	S3 (%)	S4 (%)	S5 (%)	S6 (%)	S7(%)	S8(%)(Q1b)
**Intervention M Group**	**31** (7 refused)	**24 (77)**	17 (55)	14 (45)	13 (42)	12 (39)	12 (39)	12 (39)	**19 (61)**
**Intervention F Group**	**19** (5 refused)	**14 (74)**	13 (68)	7(37)	11(58)	7 (37)	9 (47)	6 (32)	**12 (63)**
**Total**	**50**	**38 (76)**	30 (60)	21 (42)	24 (48)	19 (38)	21 (42)	18 (36)	**31 (62)**
**Control M Group**	**30** (12refused)	**18 (60)**	9 (30)	8 (27)	6 (20)	4 (13)	5 (17)	4 (13)	**9 (30)**
**Control F Group**	**21** (5 refused)	**16(76)**	13 (62)	12 (57)	11 (52)	13 (62)	13 (62)	10 (48)	**15 (71)**
**Total**	**51**	**34(67)**	22 (43)	20 (39)	17 (33)	17 (33)	18 (35)	14 (27)	**24 (47)**

Note: S= PAAI sessions, Q1a= questionnaire at first session, Q1b= questionnaire at last session, M= men group, and F= female group.

Supplementary Table S2. **Group comparisons on characteristics of follow-up and dropout**

		Follow-up	Dropout	P-value
Total		65	36	
Age (years), Mean (SD)		37.8 (10.3)	34.0 (12.8)	0.10
Low health literacy, *n* (%)		36 (55)	14 (39)	0.11
Female, *n* (%)		28 (43)	12 (33)	0.33
Ethnicity, *n* (%)	Arab	48 (74)	29 (81)	0.61
	Kurd	16 (25)	7 (19)	
Stayed in a transit country on way to Norway *n* (%)		46 (71)	14 (39)	0.002
Marital status (married), *n* (%)		42 (65)	20 (56)	0.37
Have children, *n* (%)		48 (74)	20 (56)	0.06
Number of children, Mean (SD)		3.4 (1.5)	3.2 (1.3)	0.55
Education (years), Mean (SD)		8.6 (4.2)	11.4 (3.9)	0.002
Self-reported health, *n* (%)				
	Poor	31(48)	10(28)	0.15
	Neither	22 (34)	17 (47)	
	Good	1218	9 (25)	
Self-reported diseases and daily use of medication, *n* (%)				
Physical or psychological pain at least 1year		41 (63)	19 (53)	0.31
Physical pain more >6 months		50 (77)	25 (70)	0.41
Never do exercise		38 (58)	11 (31)	0.007
Rheumatic arthritis		13 (20)	6 (17)	0.68
Joint disease		48 (74)	23 (64)	0.29
Mental health problems		8 (12)	5 (14)	0.82
Headache		22 (34)	7 (19)	0.12
Daily use of painkillers		19 (29)	8 (22)	0.73
Daily use of psychotropics		8 (12)	2 (6)	0.09
Study outcomes				
Impact events scales revised IESR, Mean (SD)	Intrusion (8-32)	9.4 (7.6)	9.3 (7.9)	0.96
	Avoidance (8-32)	9.5 (7.9)	10.7 (7.9)	0.46
	Hyper-arousal (6-24)	7.4 (5.7)	7 (5.8)	0.74

BPI scores	Having pain today (yes), *n* (%)	65 (100)	35 (97)	0.17
	Pain intensity (1-10), Mean (SD)	6.0 (1.9)	5.4 (2.3)	0.21
GHQ-12 (0-36), Mean (SD)		13.4 (6.1)	11.2 (6.7)	0.10

Supplementary Table S3. Change in outcomes from first to last sessions and four weeks after last session for intervention and control groups combined (*n* = 101) using liners mixed models

		Week 0	Week 8		Week 12		P-trend
			B (85% CI)	p-value	B (85% CI)	p-value	
**Total**							
**BPI**		0 (ref)	-0.8 (-1.2, -0.5)	<0.001	-1.0 (-1.4, -0.6)	<0.001	<0.001
**IES-R**		0 (ref)	0.6 (-2.5, 3.7)	0.71	-0.5 (-3.7, 2.8)	0.78	0.90
**GHQ**		0 (ref)	-2.1 (-3.3, -0.9)	<0.001	-2.3 (-3.6, -1.1)	<0.001	<0.001
**Women**							
**BPI**		0 (ref)	-1.0 (-1.6, -0.5)	<0.001	-1.1 (-1.7, -0.5)	<0.001	<0.001
**IES-R**		0 (ref)	-2.8 (-6.8, 1.2)	0.17	-2.3 (-6.5, 1.9)	0.28	0.20
**GHQ**		0 (ref)	-2.0 (-3.6, -0.4)	0.02	-1.9 (-3.6, -0.2)	0.03	0.01
**Men**							
**BPI**		0 (ref)	-0.7 (-1.2, -0.2)	0.008	-0.9 (-1.4, -0.4)	0.001	<0.001
**IES-R**		0 (ref)	3.6 (-0.9, 8.2)	0.12	0.9 (-3.7, 5.6)	0.70	0.44
**GHQ**		0 (ref)	-2.1 (-3.8, -0.3)	0.02	-2.6 (-4.4, -0.8)	0.005	0.002

Note: P-valued for interaction tests by gender: BPI: 0.69, IES-R: 0.13 and GHQ: 0.85.
